# In Situ *Fusarium* Mycotoxin Mitigation During Wholegrain Wheat Flour Fermentation with Sourdough Lactic Acid Bacteria and Further Insights into Reduction Mechanisms

**DOI:** 10.3390/foods15193543

**Published:** 2026-10-04

**Authors:** Giuseppe Perri, Claudia Sardella, Lorenzo Ciraldo, Valentina Scarpino, Simona Carbone, Federico Rametta, Massimo Blandino, Carlo Giuseppe Rizzello, Erica Pontonio

**Affiliations:** 1 Department of the Soil, Plant and Food Science (DiSSPA), University of Bari Aldo Moro, Via Amendola, 165/a, 70126 Bari, Italy; lorenzo.ciraldo@uniba.it (L.C.); simona.carbone@uniba.it (S.C.); federico.rametta@uniba.it (F.R.); erica.pontonio@uniba.it (E.P.); 2 Department of Agricultural, Forest and Food Sciences, University of Turin, Largo Paolo Braccini 2, 10095 Grugliasco, Italy; claudia.sardella@unito.it (C.S.); valentina.scarpino@unito.it (V.S.); massimo.blandino@unito.it (M.B.); 3 Department of Environmental Biology, “Sapienza” University of Rome, 00185 Rome, Italy; carlogiuseppe.rizzello@uniroma1.it

**Keywords:** lactic acid bacteria, sourdough, wholegrain wheat, deoxynivalenol, enniatins, moniliformin, food safety

## Abstract

*Fusarium* mycotoxins are major contaminants of cereal-based products, yet effective clean-label mitigation strategies are still limited. Although lactic acid bacteria (LAB) have been extensively investigated as biological tools for mycotoxin reduction, evidence is largely based on in vitro studies. Here, nineteen LAB strains representative of sourdough microbiota were screened for their ability to reduce deoxynivalenol (DON) in wholegrain wheat dough. Pronounced strain-dependent differences were observed, leading to the selection of *Lactiplantibacillus plantarum* 1A7, *Limosilactobacillus fermentum* 10B, and *Pediococcus pentosaceus* I76 for further evaluation against a broader panel of *Fusarium* regulated and emerging mycotoxins, including modified DON derivatives, moniliformin, and enniatins. After 48 h of fermentation, with LAB populations reaching approximately 9 log CFU g^−1^, the selected strains achieved comparable reduction efficiencies across multiple analytes, decreasing mycotoxin levels by approximately 50% and lowering DON concentrations from 1270 µg kg^−1^ to below the European regulatory limit of 600 µg kg^−1^ for cereal milling products. To gain insight into the mechanisms involved, fermentations were performed using both viable and heat-inactivated cells. The lower reduction efficiency observed with inactivated cells was consistent with a contribution of cell wall-mediated adsorption, although additional contributions from LAB metabolic activity cannot be excluded. Overall, sourdough LAB effectively reduced multiple mycotoxins in a complex cereal matrix, with the observed effects indicating a reduction in extractable mycotoxin levels without providing conclusive evidence of detoxification. These findings provide new insights into LAB–mycotoxin interactions and support fermentation-based strategies for mycotoxin mitigation in wheat-based foods.

## 1. Introduction

In recent years, rapid environmental changes and rising global temperatures have increasingly challenged ecosystems, agricultural productivity, and food safety [1,2]. Climate change, in particular, can promote fungal growth and stimulate mycotoxin production by altering temperature, humidity, and precipitation patterns [3,4,5]. To date, more than 400 toxic fungal secondary metabolites have been identified, and mycotoxins are recognized as widespread contaminants of agricultural commodities worldwide [6]. These compounds are associated with a wide range of adverse health effects, including immunotoxicity, nephrotoxicity, carcinogenicity, and developmental disorders [7]. Wheat (*Triticum* ssp.) represents one of the most important staple crops globally, contributing about 20% of daily protein and caloric intake [8]. However, it is also particularly vulnerable to colonization by toxigenic fungi. Mycotoxin accumulation in wheat is influenced by several interacting factors, including fungal species, grain maturity, pest pressure, environmental conditions such as temperature, water activity, and drought stress [9]. Given its widespread use in staple foods like bread and pasta, wheat is considered a major source of dietary exposure to mycotoxins, especially deoxynivalenol (DON) [10,11]. To limit potential health risks, maximum levels (MLs) for major mycotoxins in cereals and cereal-based products, including DON and zearalenone (ZEA), have been established by international authorities. In the European Union, these limits are established under Commission Regulation (EU) 2023/915, as amended [12,13], and vary according to the type of cereal commodity and cereal-based product. In addition to these regulated compounds, cereals may also contain so-called emerging or modified mycotoxins, such as DON conjugates (3- and 15-acetyl-deoxynivalenol, 3-ADON and 15-ADON), deoxynivalenol-3-glucoside (DON-3-G), moniliformin (MON), nivalenol (NIV), and enniatins (ENN_TOT_). These compounds are increasingly reported in European cereals; however, they remain poorly studied and currently lack specific regulatory frameworks [14,15]. A range of mitigation strategies has been proposed to control mycotoxin contamination, targeting both pre-harvest and post-harvest stages. Agronomic practices such as crop rotation and soil tillage, the use of tolerant cultivars, fungicide application, and integrated disease management can help reduce fungal development, although their effectiveness may be limited under high-risk environmental conditions [16]. Post-harvest interventions and food-processing technologies can further contribute to reducing contamination through physical, chemical, or enzymatic approaches [17]. However, these strategies are often costly, may negatively affect product quality, or are not fully aligned with the growing demand for clean-label foods [18]. Moreover, according to Regulation (EU) 2023/915 [13], food products exceeding the established maximum levels cannot be rendered compliant through chemical detoxification treatments. Therefore, increasing attention has been directed towards preventive and biological mitigation strategies aimed at reducing mycotoxin contamination levels during production and processing while maintaining food safety and quality. In this context, biopreservation based on selected microorganisms has emerged as a promising alternative for improving food safety [19,20]. Among these, lactic acid bacteria (LAB) are particularly interesting due to their long history of safe use in food fermentation and their recognized GRAS/QPS status [21]. LAB can inhibit spoilage microorganisms and fungal growth through the production of natural antimicrobial compounds, making them suitable candidates as “green” preservatives [22]. More recently, increasing attention has been paid to the potential of LAB to reduce mycotoxin levels in sourdough systems, including both defined starter cultures and complex microbial consortia typical of spontaneous fermentations [23,24]. However, most studies investigating LAB-mediated mycotoxin reduction have been conducted under in vitro conditions, often using simplified culture media-based assays, which may not fully reflect the complexity of cereal matrices. Consequently, despite increasing evidence of LAB potential as biological mitigation agents, the mechanisms underlying mycotoxin reduction remain not fully elucidated and may involve different processes, including adsorption to microbial cell surfaces, enzymatic biotransformation, and interactions with fermentation-derived metabolites [25,26,27]. In addition, in situ studies remain relatively limited, typically focusing on a small number of LAB species and primarily addressing parent mycotoxins. Only a few studies have considered degradation products or emerging mycotoxins such as MON and ENN_tot_ [28,29]. Against this background, the present study aimed to evaluate the potential of sourdough-associated LAB strains for in situ mitigation of *Fusarium* mycotoxins during fermentation of naturally contaminated wholegrain wheat flour, with particular attention to DON, its modified forms, and emerging *Fusarium* mycotoxins such as moniliformin and enniatins. The study also aimed to provide further insights into the mechanisms underlying LAB-mediated mycotoxin reduction.

## 2. Materials and Methods

### 2.1. Experimental Rationale

The experimental workflow was designed to evaluate the potential of LAB fermentation to mitigate *Fusarium* mycotoxins in wholegrain wheat flour obtained from naturally contaminated wheat. The study consisted of two main experimental stages.

(i) LAB screening and fermentation optimization. Nineteen sourdough-associated LAB strains, belonging to species commonly encountered in sourdough fermentations and previously investigated for their technological potential and ability to mitigate chemical contaminants in food systems [30,31,32,33], were included in the study. The strains comprised autochthonous isolates belonging to the culture collection of the Department of Soil, Plant and Food Sciences (DiSSPA), University of Bari Aldo Moro, obtained from different types of sourdough and identified at species level by PCR amplification and Sanger sequencing of the *16S rRNA* gene, followed by BLAST analysis against GenBank, as previously described by Pontonio et al. (2025) [34]. For each species represented by the autochthonous isolates, a corresponding strain from the DSMZ culture collection was included as a type strain for comparison. They were screened for their ability to reduce DON during wholegrain wheat dough fermentation. Based on DON reduction performance, the most effective strain–process combinations were selected for further investigation.

(ii) Mechanistic investigation and multi-mycotoxin characterization. The selected strain–process combinations were further investigated using fermentations performed with viable and heat-inactivated cells to assess the relative contributions of microbial activity and cell-associated mechanisms to extractable mycotoxin reduction. The resulting fermentation samples were simultaneously analysed by LC–MS/MS to quantify DON, its modified forms, and additional emerging *Fusarium* mycotoxins, providing a comprehensive characterization of fermentation-induced changes in the mycotoxin profile.

### 2.2. Wheat Cultivation and Milling

Bread wheat was cultivated in the 2023–24 growing at the experimental farm of the University of Turin in Carmagnola (Northwest Italy). The crop was managed according to the standard agronomic practices of the growing area, except that no fungicide treatments were applied to control Fusarium Head Blight (FHB) at flowering, allowing natural infection by *Fusarium* species responsible for the accumulation of the major mycotoxins in the grain. Briefly, a representative ordinary bread-making cultivar (Aubusson; Limagrain Italia S.p.A., Busseto, Italy) was sowed on November 8th after an autumn plowing, with maize as the previous crop. A fungicide mixture (prothioconazole and pydiflumetofen; Miravis Pro, Syngenta Italia, Milan, Italy) was applied at booting stage (growth stage 45, according to BBCH scale, [35]) to control foliar diseases, while to minimize the FHB and DON contamination, no fungicide was applied at flowering. Wheat was harvested on July 18th with a Walter Wintersteiger (Wintersteiger, Ried im Innkreis, Austria) combine-harvester. Harvested kernels were thoroughly mixed, and a 4-kg kernel sample was collected. Naturally infected wheat kernels were ground to wholegrain flour using a centrifugal mill equipped with a 1-mm sieve (ETM mill, Vercella Fabio, Mercenasco, Italy) prior to mycotoxin analysis and LAB fermentation.

### 2.3. LAB Strains and Growth Conditions

LAB strains belonging to the species *Lactiplantibacillus plantarum* (1A7, CR1, M7S2, A1, DSM 1055), *Limosilactobacillus fermentum* (10B, La5, F230, DSM 20049, DSM 20052), *Pediococcus pentosaceus* (I76, T1C1, CISEW5, 13I, DSM 20336), and *Levilactobacillus brevis* (P7m1, MRS4, MCL-2, DSM 1269) were cultured in MRS medium (Oxoid, Basingstoke, UK) at 30 °C for 16 h. Cells were harvested by centrifugation (10,000× *g*, 10 min, 4 °C), washed twice with 50 mM phosphate buffer (pH 7.0), and resuspended in tap water prior to dough preparation.

### 2.4. Dough Preparation and Fermentation Monitoring

Wholegrain wheat doughs (200 g) were prepared by mixing flour and water at a 1:1 (*w*/*w*) ratio. Each dough was inoculated with an individual LAB strain to achieve an initial cell density of 7 log CFU g^−1^. Fermentation trials were conducted separately for each strain at 25, 30, and 37 °C for 24 and 48 h in laboratory incubators, without active control of environmental humidity. Dough samples collected immediately after preparation and before fermentation were designated as UD (unfermented dough) and used for microbiological and acidification analyses, whereas unfermented wholegrain wheat flour samples were designated as UF (unfermented flour) and used as the reference material for mycotoxin determination.

Two control conditions were included: (i) spontaneously fermented doughs without LAB inoculation (SF), to evaluate the contribution of the endogenous microbiota to changes in mycotoxin concentrations during fermentation; and (ii) doughs supplemented with chloramphenicol and cycloheximide (0.1 g kg^−1^ dough each) without LAB inoculation (SFa), to inhibit microbial growth and estimate the contribution of endogenous non-microbial factors, including wheat enzymatic activity, to changes in mycotoxin concentrations.

LAB cell density was determined after 24 and 48 h of fermentation by plate counting on MRS agar supplemented with cycloheximide (0.1 g L^−1^) after incubation at 30 °C for 48 h. pH was measured using a Crison 507 pH meter (Milan, Italy), while total titratable acidity (TTA) was expressed as the volume (mL) of 0.1 M NaOH required to adjust 10 g of dough suspended in 90 mL of sterile water to pH 8.4 [36]. Lactic and acetic acid concentrations were quantified using K-DLATE and K-ACET enzymatic kits (Megazyme, Bray, Ireland), following the manufacturer’s instructions.

### 2.5. Preliminary Screening for Deoxynivalenol (DON) via Enzyme-Linked Immunosorbent Assay (ELISA)

Following the fermentation trials described in Section 2.4, fermented dough samples and the corresponding control conditions were subjected to preliminary DON screening to identify the LAB strain–process combinations showing the highest DON reduction capacity. DON levels were determined using a commercial ELISA kit (Elabscience Biotechnology Co., Ltd., Wuhan, China; DON ELISA Kit, Cat. No. E-TO-E011). According to the manufacturer, the kit has a detection limit of 200 μg kg^−1^ for grain and feed samples, a sample recovery of 85 ± 15%, and cross-reactivity of 100% for DON and <1% for 3-acetyldeoxynivalenol. Briefly, fermented dough samples were dried at 50 °C for 24 h. Two grams of each dried sample were homogenized, extracted with 20 mL of deionized water, vortexed for 5 min, and centrifuged at 4000 rpm for 10 min at room temperature. The supernatants were then analysed following the kit protocol. Samples were analysed in triplicate.

### 2.6. Investigation of the Mechanisms Underlying LAB-Mediated Mycotoxin Reduction

Based on the preliminary screening, the three most effective strain–fermentation condition combinations, in terms of DON reduction, were selected (*Lp. plantarum* 1A7 at 30 °C, *Lim. fermentum* 10B at 25 °C, and *P. pentosaceus* I76 at 25 °C), all after 48 h of fermentation. Doughs were prepared using wholegrain wheat flour and water at a 1:1 (*w*/*w*) ratio and inoculated with each strain at an initial density of 7 log CFU g^−1^. Fermentation was carried out under the selected conditions. In parallel, doughs were prepared with heat-inactivated cells of the same strains to investigate the contribution of non-viable biomass to mycotoxin reduction. Cells harvested after 16 h of growth (exponential phase) were heat-inactivated at 121 °C for 15 min, collected by centrifugation, and washed twice with 50 mM phosphate buffer (pH 7.0). The resulting inactivated cells were inoculated into the dough at an estimated cell density of 9 log CFU g^−1^. Cell density was estimated by measuring optical density at 620 nm (OD_620_), where an OD_620_ value of 2.5 corresponded to approximately 9.0 log CFU mL^−1^. Doughs containing inactivated cells were supplemented with chloramphenicol and cycloheximide (0.1 g L^−1^ each) and incubated under the same time–temperature conditions applied to the corresponding viable strains. Microbial growth and acidification parameters (pH, TTA, lactic acid, and acetic acid concentrations) were determined as described in Section 2.4. Following fermentation, samples intended for mycotoxin analysis, including the control samples (SF and SFa) described in Section 2.4, were dried at 50 °C for approximately 24 h in a ventilated oven (Binder, Germany) until a moisture content comparable to that of native flour was reached.

### 2.7. Determination of DON, DON Modified Form, and Emerging Mycotoxins by HPLC-MS/MS

Mycotoxin analysis was performed in triplicates on naturally infected wholegrain flours and fermented doughs using a multi-mycotoxin liquid chromatography–tandem mass spectrometry (LC–MS/MS) method as described by Scarpino et al. (2019) [37]. The following mycotoxins were simultaneously determined in each sample: deoxynivalenol (DON), deoxynivalenol-3-glucoside (DON-3-G), 3-acetyldeoxynivalenol (3-ADON), 15-acetyldeoxynivalenol (15-ADON), and total deoxynivalenol forms (DONTOT) as the sum of DON, DON-3-G, 3-ADON and 15-ADON; nivalenol (NIV), zearalenone (ZEA), moniliformin (MON), enniatin A (ENN A), enniatin A1 (ENN A1), enniatin B (ENN B), enniatin B1 (ENN B1), and total enniatin forms (ENNTOT) as the sum of ENN A, A1, B and B1. Briefly, 5.0 g of each wholegrain flour was extracted with 20 mL of CH_3_CN/H_2_O/CH_3_COOH (79/20/1, *v*/*v*/*v*), according to the dilute-and-shoot method reported by Scarpino et al. (2019) [37], and 20 µL of the diluted filtered extracts was analysed without any further pre-treatment on a Varian 310 triple quadrupole mass spectrometer (Varian, Milan, Italy), equipped with an electrospray ionization (ESI) source, a 212 LC pump, a ProStar 410 AutoSampler, and dedicated software. Liquid chromatography (LC) separation was performed on a Gemini-NX C18 100 × 2.0 mm i.d., 3 µm particle size, 110 Å, equipped with a C18 4 × 2 mm security guard cartridge column (Phenomenex, Torrance, CA, USA). The mobile phase consisted of two eluents (VWR, Milan, Italy), H_2_O (eluent A) and CH_3_OH (eluent B), both of which were acidified with 0.1% *v*/*v* CH_3_COOH (Sigma-Aldrich, St. Louis, MO, USA) and delivered at 200 mL min^−1^. In order to quantify all the analytes with positive and negative polarity, two separate chromatographic runs per sample were carried out. The run for the negative ionization mode acquisition took 15 min and consisted of the following gradient: after an initial time of 2 min at 90% A, the proportion of B was increased linearly to 100% in 2 min, and this was followed by a hold time of 2 min at 100% B and 4 min column re-equilibration at 90% A; finally, the initial condition at 90% A was kept for 5 min before the subsequent chromatographic run. The positive ionization mode acquisition run instead took 13 min and consisted of the following gradient: the elution was started with 90% A and the proportion of B was increased linearly over 2 min to 100%; this condition was kept for 2 min and B was then decreased linearly to the initial condition of 10% over 5 min and kept at this level for 4 min in order to re-equilibrate the column. Mass spectrometric analysis (ESI-MS/MS) was performed, in selected reaction monitoring (SRM) mode, alternating two transition reactions for each compound in both negative and in positive ionization modes in two separate chromatographic runs per sample with the following settings: the nebulizing gas was N2 (20 psi); the drying gas was air (250 °C in negative ionization mode and 300 °C in positive ionization mode, 25 psi); the needle voltage was –4000 V and +5000 V, the shield voltage was –600 V and +600 V and the detector voltage was –1950 V and +1950 V, for negative and positive polarity, respectively; and the collision gas was Ar (2.00 mTorr). Table S1 summarizes the optimized SRM transition parameters for the analyzed mycotoxins. Mycotoxin standards were purchased from different sources and were dissolved in CH3CN, if not stated otherwise. Two composite standard working solutions were prepared by dissolving appropriate volumes of each analyte in a diluting phase mixture, as specified in Scarpino et al. (2019) [37]. These two working solutions were then mixed in appropriate volumes and dissolved in CH_3_CN/H_2_O/CH_3_COOH 49.5/49.5/1 *v*/*v*/*v* in order to prepare the working solutions for the calibration. A set of three different calibration curves was prepared in triplicate: an external calibration in neat solvent, a matrix-matched calibration curve, and a matrix-matched calibration curve in sample extracts. The apparent recovery (%), the signal suppression/enhancement due to the matrix effects and recovery of extraction were calculated from the calibration curves, as specified in Scarpino et al. (2019) [37]. The results of the concentrations of mycotoxins and other metabolites quantified were corrected for the recovery rates and expressed as µg kg^−1^ on dry matter basis. The limits of detection (LOQ) were as follows: 4.2 µg kg^−1^ for DON, 10 for DON-3-G, 3-ADON, 15-ADON, and NIV; 1.2 µg kg^−1^ for ZEA; 1.6 µg kg^−1^ for ENN A; 1.8 for ENN A1; 1.4 µg kg^−1^ for ENN B; and1.5 µg kg^−1^ for ENN B1.

### 2.8. Statistical Analysis

Data normality and homogeneity of variance were assessed using the Shapiro–Wilk and Bartlett’s tests, respectively, implemented in R software (version 3.2.3). Data were analysed by one-way analysis of variance (ANOVA) followed by Tukey’s honestly significant difference (HSD) post-hoc test. Statistical significance was established at *p*-value ≤ 0.05. For the screening based on DON reduction experiments, separate analyses were conducted for each fermentation temperature (25, 30, and 37 °C), including all treatments and fermentation times within the same dataset. For microbial counts and acidification parameters, analyses were performed separately for each fermentation time using treatment as the main factor. All experiments were conducted using three independent biological replicates, and results are expressed as mean ± standard deviation.

## 3. Results and Discussion

### 3.1. Selection of Best Performing LAB Strains and Fermentation Parameters

**Figure 1:**
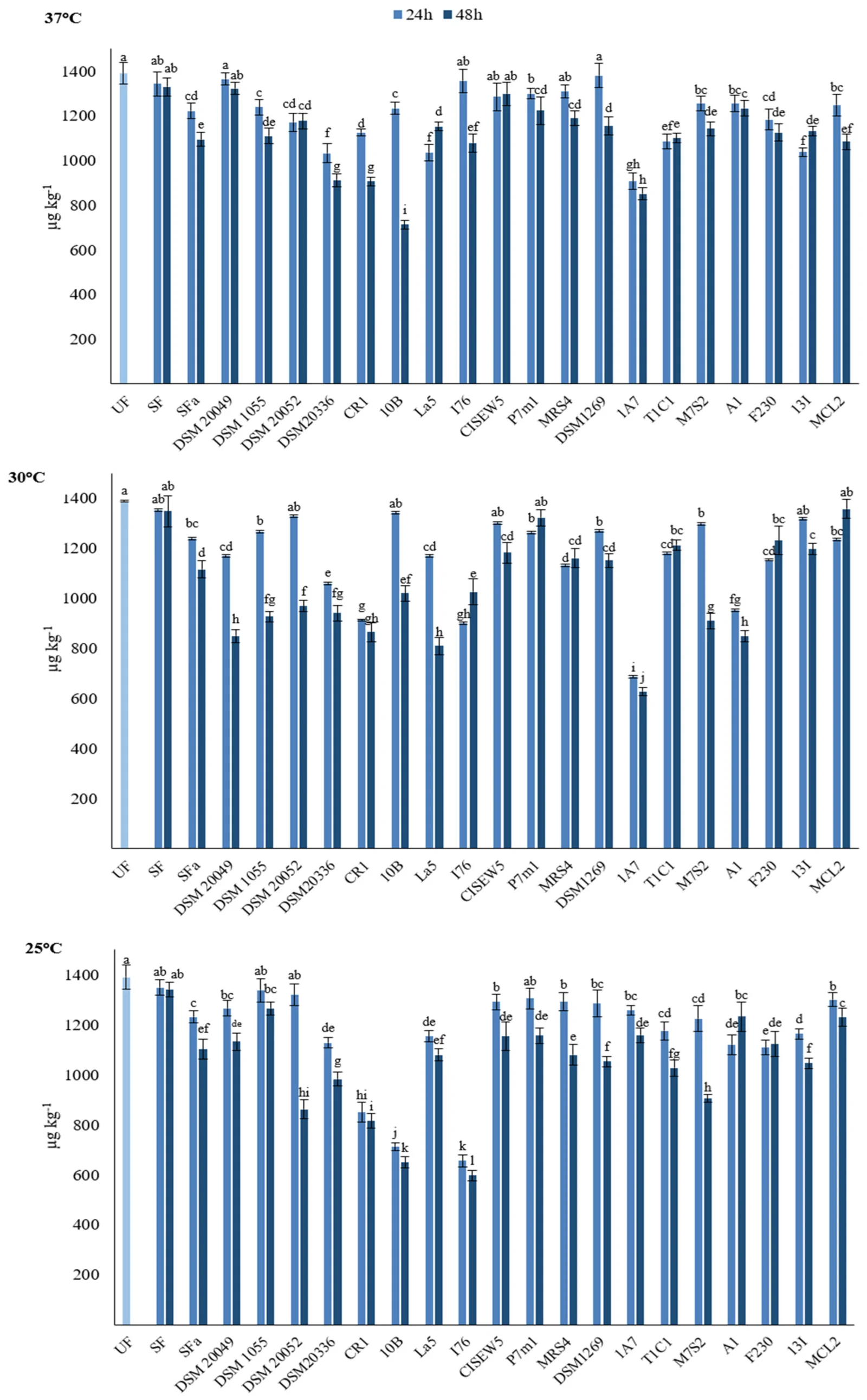
Effect of fermentation with selected lactic acid bacteria (LAB) on deoxynivalenol (DON) concentrations (µg kg^−1^) in wholegrain wheat flour. Fermentation was carried out for 24 and 48 h at 25, 30, and 37 °C in 1:1 flour–water doughs using 19 LAB strains: *Lactiplantibacillus plantarum* (1A7, LB1, M7S2, A1, DSM 1055), *Limosilactobacillus fermentum* (10B, La5, F230, DSM 20049, DSM 20052), *Pediococcus pentosaceus* (I76, T1C1, CISEW5, 13I, DSM 20336), and *Levilactobacillus brevis* (P7m1, MRS4, MCL-2, DSM 1269). Controls included unfermented wholegrain wheat flour (UF), spontaneously fermented doughs (SF), and doughs supplemented with chloramphenicol and cycloheximide without starter inoculation (SFa). Bars represent mean ± standard deviation; different letters indicate significant differences among treatments and fermentation times according to Tukey’s test (*p* < 0.05).

DON is the most prevalent *Fusarium* mycotoxin in cereals such as wheat, oats, and barley [38]. Several studies have reported the ability of lactic acid bacteria (LAB) to reduce mycotoxins, although most evidence derives from simplified in vitro systems [27,39,40]. Reduction efficiency depends on factors such as toxin concentration, cell density, strain characteristics, matrix complexity, and fermentation conditions [41,42]. DON is considered particularly recalcitrant to LAB-mediated reduction [41] and was therefore selected as a model compound for strain screening and process optimization. As reported in Tables S2–S4, all strains grew efficiently at 25, 30, and 37 °C, reaching final cell densities of 8.1–9.8 log CFU g^−1^ (≈2–3 log increase vs. UD). Growth was accompanied by marked acidification (pH 6.3 to 3.3–4.4 and increased total titratable acidity (TTA), with organic acid production reaching up to 154 mmol kg^−1^ for lactic acid and 75 mmol kg^−1^ for acetic acid, particularly after 48 h of fermentation. These results are consistent with the typical behavior of LAB in wheat-based matrices [43]. As shown in Figure 1, fermentation led to a reduction in DON concentration compared with UF under all conditions, although the decreases were not always statistically significant and were influenced by both time and temperature. After 24 h, reductions averaged ~16%, while after 48 h, both the extent of reduction and inter-strain variability increased, reaching up to 24.6% at 25 and 30 °C and ~20% at 37 °C. Previous studies evaluating panels of LAB strains report variable DON reduction, ranging from ≤10% to 30–50% [39,41,44]. Notably, highly performing outlier strains were identified. Strains I76 (*P. pentosaceus*) and 10B (*Lim. fermentum*) achieved reductions of 57% and 53%, respectively, at 25 °C after 48 h, while strain 1A7 (*Lp. plantarum*) reached 54% at 30 °C after 48 h (Figure 1). These values align with the range of the highest DON reductions reported in wheat-based fermentation systems (44–69%) [28,29], involving species such as *Lp. plantarum*, *P. acidilactici*, *Lacticaseibacillus casei*, *Lact. paracasei*, and *Liquorilactobacillus uvarum*. SF showed negligible DON reduction under all conditions, whereas SFa consistently exhibited a decrease of approximately 20% after 48 h at all tested temperatures (Figure 1).

Although this was not experimentally verified in the present study, the observed reduction may be related to endogenous wheat enzymatic activity, such as that of peroxidases and lipoxygenases, which under relatively stable pH conditions may contribute to mycotoxin modification [45,46]. Trakselite-Rupsiene et al. [29] further reported correlations between amylolytic and xylanolytic activities and DON reduction in wheat doughs, supporting this hypothesis. In SF, however, acidification and microbial activity may limit this effect, as also reported in similar systems [47]. Three main mechanisms have been proposed to explain microbial mycotoxin removal: enzymatic degradation, adsorption to microbial cells, and interactions with fermentation-derived metabolites [48]. For LAB-mediated DON reduction, adsorption to the cell wall is generally considered the predominant mechanism [39,49]. The complex structure of the cell wall, composed of peptidoglycan, teichoic acids, polysaccharides, and proteins, provides multiple binding sites enabling toxin sequestration [50]. However, adsorption is typically characterized by rapid kinetics, occurring within the first hours of contact, with most binding established early and minimal changes thereafter [51,52,53,54]. Although mycotoxin enzymatic detoxification is typically associated with non-LAB bacteria, particularly *Bacillus* spp., some LAB species, including *Lev. brevis* and *Lp. plantarum*, have been shown to be involved in DON biotransformation [26,49,55]. Therefore, the observed variability, along with the effects of temperature and incubation time, led to the hypothesis that additional strain-dependent mechanisms beyond adsorption may be involved.

### 3.2. Investigation of the Mechanisms Underlying Mycotoxin Reduction

To elucidate the mechanisms underlying mycotoxin reduction, the most effective LAB strains were inoculated into wheat doughs in both active and heat-inactivated forms, adapting previously established in vitro methods [51,52,56] to an in situ wheat dough model system. In line with previous findings indicating that adsorption capacity is strongly influenced by LAB cell density [26], heat-inactivated cells were inoculated at 9 log CFU g^−1^, corresponding to the final cell density reached by viable LAB at the end of fermentation (Table 1).

LC–MS/MS analysis extended the investigation beyond DON to include DON-3-G, 3-ADON, 15-ADON, DON_TOT_, ZEA, and emerging mycotoxins such as NIV, all produced by *F. graminearum* and *F. culmorum*, and MON and ENN_TOT_, mainly produced by *F. avenaceum* (Table 2).

The levels of 15-ADON, ZEA, NIV, ENN A, and ENN A_1_ were < LOQ for the naturally contaminated unfermented flour (UF), while the detected metabolites included DON, DON-3-G, 3-ADON, MON, ENN B, and ENN B_1_. DON, the only regulated mycotoxin detected, was the most prevalent and was found at a concentration of 1270 µg kg^−1^ in the naturally contaminated unfermented flour (UF), exceeding the regulatory limit of 600 µg kg^−1^ established by EU Regulation 2024/1022 for cereal milling products. The occurrence of high DON levels highlights the relevance of crop management practices in determining the initial contamination level of wheat grains. Previous studies have shown that preventive measures within intensive cereal systems, such as fungicide application at flowering to control FHB, can substantially affect DON accumulation, with reductions in DON and other co-occurring *Fusarium* spp. metabolites of up to 73% [57].

Fermentation with selected LAB strains resulted in significant reductions in multiple mycotoxins compared with UF (Table 2), with limited inter-strain variability; however, *P. pentosaceus* I76 consistently showed the highest overall performance across compounds. In viable fermentations, DON decreased from 1270 µg kg^−1^ in UF to 567–680 µg kg^−1^ (>50% reduction), consistent with preliminary screening. Comparable reductions were observed for DON-3-G and 3-ADON, reaching up to 53% and 39%, respectively (Table 2). Heat-inactivated cells also induced reductions, although with consistently lower efficiencies than viable cells. Similar trends were observed for enniatins (ENN B and ENN B_1_), with reductions up to ~50% in active fermentations and 10–25% in heat-inactivated systems (Table 2). These results are consistent with previous in situ wheat fermentation studies [28]. To the best of the authors’ knowledge, despite its proven toxicological relevance [58], few studies have attempted to investigate MON reduction through LAB fermentation. Here, MON significantly decreased from 106 µg kg^−1^ in UF to 45.4–57.3 µg kg^−1^ in active fermentations and to 76.9–89.9 µg kg^−1^ in heat-inactivated treatments. Overall, these findings support adsorption as a major contributing mechanism responsible for mycotoxin reduction.

The lower efficiency observed in heat-inactivated cells can be attributed to structural and physicochemical alterations of the cell wall that reduce binding capacity [59,60]. Conversely, fermentation-associated acidification may enhance adsorption by increasing cell surface hydrophobicity and modifying functional groups on the cell wall [51,61,62]. Importantly, adsorption appeared largely independent of toxin-specific physicochemical properties known to influence binding processes ([63]; Table S5), as similar reductions were observed across structurally diverse compounds, ranging from the highly polar MON (MW 98.06 g mol^−1^; LogD −3.79) to hydrophobic enniatins such as ENN B_1_ (MW 653.86 g mol^−1^; LogD 3.81).

Although heat treatments are generally expected to reduce biological activity, the literature evidence suggests that thermal processing does not necessarily impair adsorption capacity and may even enhance binding through structural rearrangements of cell wall components, increased exposure of binding sites, and changes in secondary structure (e.g., α-helices and β-turns), ultimately increasing surface disorder and accessibility for toxin interaction [50,54,64,65,66]. In addition, a secondary contribution of LAB metabolic activity in viable cells cannot be excluded, potentially occurring after initial adsorption of toxins onto the cell surface, as previously proposed [50].

Within the limits of the present dataset (Table 1 and Table 2), and considering the enzymatic repertoire of LAB [67,68,69] together with the chemical structure of the detected mycotoxins (Table S5), potential biotransformation reactions may include hydrolysis of DON-3-G and deacetylation of 3-ADON, leading to free DON release. However, the stability of the DON-to-masked form ratios (DON-3-G/DON and 3-ADON/DON, Table 2) across treatments compared with UF may be consistent with limited net accumulation of DON during these potential transformation reactions. However, this observation alone does not allow the predominant contribution of adsorption to be established. Indeed, other possibilities cannot be excluded, including the re-adsorption of transiently released DON onto bacterial cell surfaces or its conversion into non-quantified, less toxic metabolites. Supporting the latter possibility, alternative LAB-mediated transformation pathways, such as epimerization to 3-epi-DON, have been reported in *Lac. rhamnosus* strains [70]. Regardless of the mechanism involved, the observed reduction should not be interpreted as definitive evidence of detoxification. If adsorption contributes to the observed reduction, changes in gastrointestinal pH and physicochemical conditions may affect toxin–cell interactions and the stability of mycotoxin sequestration during gastrointestinal transit [71,72].

## 4. Conclusions

LAB- and sourdough-based approaches have shown strong potential as biotechnological strategies for reducing mycotoxin contamination. In this context, the present study provides a systematic preliminary in situ evaluation combining representative sourdough LAB species with a broad spectrum of *Fusarium* mycotoxins. Three selected starter cultures in their metabolically active form significantly reduced initial DON levels (>1000 µg kg^−1^), resulting, for one of the strains, in final concentrations below the regulatory limit (<600 µg kg^−1^) established by EU Regulation 2024/1022 for milling products of cereals. The present study demonstrates a reduction in extractable mycotoxin levels rather than definitive evidence of complete detoxification. Consequently, the possible presence of non-quantified transformation products or masked metabolites with different toxicological properties cannot be excluded. The similar reduction patterns observed for DON and other *Fusarium* mycotoxins under the same fermentation conditions represent an interesting preliminary observation that warrants further investigation to determine whether DON reduction could be used as an indirect indicator of broader changes in the mycotoxin profile during LAB fermentation. However, the currently available data do not allow this relationship to be established. Moreover, although the most effective fermentation conditions identified in the present study required 48 h of incubation, such a fermentation step remains compatible with modern bakery processes because sourdough can be produced separately and subsequently used as a stable ingredient, including in the form of dried Type III sourdough preparations. Beyond their technological functionality, these preparations can serve as carriers of fermentation-derived aroma compounds and bioactive or nutritionally relevant molecules, while drying facilitates handling and storage and may reduce the contribution of volatile acidic compounds to the final sensory profile [73,74,75]. In addition, future studies should therefore validate these findings using wheat materials with different origins, cultivars, and contamination profiles, integrating metagenomic and metatranscriptomic approaches together with untargeted LC–MS/MS metabolomics to distinguish adsorption-driven reduction mechanisms from potential metabolic biotransformations, clarify the stability and mechanistic basis of LAB–mycotoxin interactions, and distinguish reversible sequestration phenomena from true detoxification pathways. Such investigations will enable further optimization of LAB-based biotechnological strategies for reducing cereal contamination and improving sustainability in the agrifood sector.

## Figures and Tables

**Table 1:** Lactic acid bacteria (LAB) cell density and acidification parameters of wholegrain wheat doughs (flour ratio 1:1) prepared from naturally contaminated wheat flour. Doughs were analysed before inoculation (unfermented dough, UD) and after 48 h of incubation under the optimal fermentation conditions identified during the preliminary screening: *Lp. plantarum* 1A7 at 30 °C, and *Lim. fermentum* 10B and *P. pentosaceus* I76 at 25 °C. LAB strains were applied as viable or heat-inactivated cells.

Treatments	LAB (log CFU g^−1^)	pH	TTA (ml NaOH)	Lactic Acid (mmol kg^−1^)	Acetic Acid (mmol kg^−1^)
UD	6.21 ± 0.33 ^b^	6.14 ± 0.18 ^a^	2.70 ± 0.11 ^d^	n.d.	n.d.
1A7-active	9.09 ± 0.20 ^a^	3.41 ± 0.08 ^c^	22.30 ± 0.49 ^b^	125.33 ± 3.99 ^a^	n.d.
1A7-inactive	n.d.	5.87 ± 0.13 ^a^	3.10 ± 0.10 ^c^	n.d.	n.d.
10B-active	9.11 ± 0.21 ^a^	3.68 ± 0.16 ^b^	26.10 ± 1.02 ^a^	94.11 ± 1.95 ^b^	56.91 ± 1.20 ^a^
10B-inactive	n.d.	5.99 ± 0.20 ^a^	3.00 ± 0.12 ^c^	n.d.	n.d.
I76-active	9.13 ± 0.36 ^a^	3.58 ± 0.15 ^bc^	22.00 ± 0.41 ^b^	131.04 ± 3.94 ^a^	9.59 ± 0.39 ^b^
I76-inactive	n.d.	5.97 ±0.16 ^a^	2.90 ± 0.13 ^cd^	n.d.	n.d.

TTA, total titratable acidity; n.d., not detected. Values are expressed as mean ± standard deviation (*n* = 3). Different superscript letters (a–d) within the same column indicate statistically significant differences (ANOVA followed by Tukey’s HSD test, *p*-value < 0.05); means sharing the same letter are not significantly different.

**Table 2:** Concentrations of *Fusarium* mycotoxins and modified forms (µg kg^−1^) in wholegrain wheat flour and dough samples. Unfermented wholegrain wheat flour (UF) was analysed as the reference material before dough preparation, whereas dough samples were analysed after 48 h of fermentation under the optimal conditions identified during the preliminary screening: *Lp. plantarum* 1A7 at 30 °C, and *Lim. fermentum* 10B and *P. pentosaceus* I76 at 25 °C. LAB strains were applied as viable or heat-inactivated cells.

Treatments	DON	DON-3-G	3-ADON	DON_TOT_	DON-3-G/ DON (%)	3-ADON/ DON (%)	MON	ENN B	ENN B_1_	ENN_TOT_
UF	1270.4 ± 84.53 ^a^	290.0 ± 17.77 ^a^	109.5 ± 5.13 ^a^	1674.9 ± 69.41 ^a^	23.0 ± 2.91 ^ab^	8.6 ± 0.80 ^b^	106 ± 3.43 ^a^	192.8 ± 4.83 ^a^	74.8 ± 2.26 ^a^	269.3 ± 13.00 ^a^
1A7-active	611.7 ± 32.12 ^de^	142.4 ± 10.62 ^f^	67.5 ± 2.05 ^e^	826.5 ± 43.41 ^f^	23.3 ± 0.77 ^b^	11.1± 0.50 ^a^	53.2 ± 1.19 ^e^	111.6 ± 5.90 ^f^	38.5 ± 2.02 ^f^	151.8 ± 7.86 ^f^
1A7-inactive	1022.1 ± 28.69 ^c^	214.2 ± 6.62 ^c^	85.6 ± 1.84 ^c^	1327 ± 28.7 ^c^	21.0 ± 0.93 ^b^	8.4 ± 0.23 ^b^	84.9 ± 4.04 ^b^	166.1 ± 6.46 ^c^	60.9 ± 2.67 ^c^	228.7 ± 9.10 ^c^
10B-active	679.8 ± 36.88 ^d^	176.5 ± 1.62 ^e^	77.4 ± 0.99 ^d^	938.6 ± 38.25 ^e^	26.0 ± 1.33 ^a^	11.4 ± 0.46 ^a^	57.3 ± 2.01 ^d^	129.4 ± 2.42 ^e^	48.1 ± 2.47 ^e^	179.2 ± 0.07 ^e^
10B-inactive	1095.8 ± 36.23 ^b^	252.2 ± 4.02 ^b^	93.4 ± 1.92 ^b^	1446.4 ± 32.84 ^b^	23.0 ± 0.99 ^b^	8.5 ± 0.45 ^b^	89.9 ± 1.71 ^b^	174.3 ± 1.64 ^b^	66.1 ± 0.75 ^b^	242.2 ± 2.22 ^b^
I76-active	567.2 ± 29.25 ^e^	119.7 ± 3.42 ^g^	59.0 ± 3.85 ^f^	750.9 ± 29.83 ^g^	21.1 ± 1.58 ^b^	10.4 ± 0.60 ^a^	45.4 ± 1.52 ^f^	101.8 ± 3.00 ^g^	32.2 ± 1.22 ^g^	135.6 ± 4.13 ^g^
I76-inactive	981.0 ± 19.37 ^c^	187.4 ± 2.73 ^d^	78.1 ± 1.13 ^d^	1251.5 ± 19.65 ^d^	19.1 ± 0.44 ^c^	8.1 ± 0.20 ^b^	76.9 ± 1.13 ^c^	151.9 ± 3.99 ^d^	55.7 ± 1.99 ^d^	209.3 ± 2.05 ^d^

Deoxynivalenol (DON), deoxynivalenol-3-glucoside (DON-3-G), 3-acetyldeoxynivalenol (3-ADON), total deoxynivalenol forms (DON_TOT_) as the sum of DON, DON-3-G, 3-ADON and 15-ADON; moniliformin (MON), enniatin B (ENN B), and enniatin B_1_ (ENN B_1_), and total enniatin forms (ENN_TOT_) as sum of ENN A, A_1_, B and B_1_. UF, unfermented dough. The DON-3-G/DON and 3-ADON/DON ratios were calculated using LC–MS/MS-derived concentrations and expressed on a mass basis as percentage values (% *w*/*w*). Values are expressed as mean ± standard deviation (*n* = 3). Different superscript letters (a–g) within the same column indicate statistically significant differences according to ANOVA followed by Tukey’s HSD post hoc test (*p*-value < 0.05); means sharing the same letter are not significantly different. 15-acetyldeoxynivalenol (15-ADON), nivalenol (NIV), zearalenone (ZEA), enniatin A (ENN A), and enniatin A_1_ (ENN A_1_) were below the limits of quantification (LOQ). LOQ are specified in Section 2.7.

## Data Availability

The original contributions presented in this study are included in the article/Supplementary Material. Further inquiries can be directed to the corresponding author.

## Supplementary Materials

The following supporting information can be downloaded at: https://www.mdpi.com/article/10.3390/foods15193543/s1. Table S1. Optimized ESI-MS and ESI-MS/MS parameters and monitored transition reactions for the analyzed mycotoxins. Table S2. Lactic acid bacteria (LAB) cell density and acidification parameters of doughs (flour:water ratio 1:1) prepared from wholegrain wheat flour obtained from naturally contaminated grain samples cultivated during the 2023–2024 growing season and fermented with selected LAB strains at 25 °C for 24 and 48 h. Table S3. Lactic acid bacteria (LAB) cell density and acidification parameters of doughs (flour:water ratio 1:1) prepared from wholegrain wheat flour obtained from naturally contaminated grain samples cultivated during the 2023–2024 growing season and fermented with selected LAB strains at 30 °C for 24 and 48 h. Table S4. Lactic acid bacteria (LAB) cell density and acidification parameters of doughs (flour:water ratio 1:1) prepared from wholegrain wheat flour obtained from naturally contaminated grain samples cultivated during the 2023–2024 growing season and fermented with selected LAB strains at 37 °C for 24 and 48 h. Table S5. Key physicochemical properties of the detected mycotoxins identified in wholegrain wheat flour. Data extracted from ChemSpider (Royal Society of Chemistry).
