# Supplementary material for: In Situ Fusarium Mycotoxin Mitigation During Wholegrain Wheat Flour Fermentation with Sourdough Lactic Acid Bacteria and Further Insights into Reduction Mechanisms
Source: Foods. 2026 Oct 4;15(19):3543. doi: 10.3390/foods15193543 (PMC13664850; doi:10.3390/foods15193543)
Supplement: Supplementary file 1 [file foods-15-03543-s001.zip › foods-4525053-supplementary.pdf]

Table S1. Optimized ESI-MS and ESI-MS/MS parameters and monitored transition reactions for the analyzed mycotoxins.

Mycotoxin	Retention time (min)	Precursor ion (*m/z*)	Adduct ion	Declustering potential (V)	Product ions[a] (*m/z*)	Collision energy (V)
3-ADON	6,02	397,3	$[M+CH_3COO]^-$	−65	336.9/306.9	−8/−13
15-ADON	3,91	339,1	$[M+H]^+$	35	137.1/339.1	12/7
DON	5,67	355,2	$[M+CH_3COO]^-$	−35	265.0/294.9	−12/−10
DON-3-G	5,55	517,3	$[M+CH_3COO]^-$	−60	427.0/457.1	−18/−10
ENN A	5,86	682,7	$[M+H]^+$	60	210.2/228.3	26/27
ENN A1	5,77	668,7	$[M+H]^+$	60	210.1/228.2	29/28
ENN B	5,43	640,7	$[M+H]^+$	60	196.2/214.2	28/27
ENN B1	5,60	654,7	$[M+H]^+$	60	196.2/214.3/210.5	28/27/26
MON	11,02	96,9	$[M-H]^-$	−40	41,1	−13
NIV	5,40	371,2	$[M+CH_3COO]^-$	−40	280.8/310.9	−13/−9
ZEA	6,84	317,1	$[M-H]^-$	−80	174.7/130.4	−24/−29

[a]Numerical value are given in the order quantifier/qualifier (/second qualifier).

Table S2. Lactic acid bacteria (LAB) cell density and acidification parameters of doughs (flour:water ratio 1:1) prepared from wholegrain wheat flour obtained from naturally contaminated grain samples cultivated during the 2023–2024 growing season and fermented with selected LAB strains at 25 °C for 24 and 48 h.

	LAB (log CFU g^{-1})		pH		TTA (ml NaOH)		Lactic acid (mmol kg^{-1})		Acetic acid (mmol kg^{-1})	
UD	6.52 ± 0.21^{d}		6.33 ± 0.16^{a}		2.80 ± 0.09^{h}		n.d.		n.d.	
	24 h	48 h	24 h	48 h	24 h	48 h	24 h	48 h	24 h	48 h
Lp. plantarum										
DSM 1055	8.90 ± 0.26^{c}	8.89 ± 0.26^{b}	3.84 ± 0.11^{cd}	3.52 ± 0.18^{cd}	19.30 ± 0.60^{b}	23.10 ± 0.37^{b}	110.17 ± 2.31^{c}	117.25 ± 3.63^{b}	2.19 ± 0.05^{m}	3.01 ± 0.09^{n}
1A7	9.79 ± 0.50^{a}	9.50 ± 0.44^{ab}	3.72 ± 0.19^{cd}	3.39 ± 0.15^{d}	13.50 ± 0.30^{f}	16.20 ± 0.60^{f}	102.34 ± 2.87^{d}	113.54 ± 3.97^{bc}	n.d.	n.d.
M7S2	9.67 ± 0.35^{ab}	9.34 ± 0.18^{ab}	3.72 ± 0.19^{cd}	3.58 ± 0.16^{cd}	17.60 ± 0.55^{cd}	23.40 ± 0.35^{b}	100.30 ± 2.31^{cd}	108.91 ± 4.47^{c}	n.d.	n.d.
CR1	9.28 ± 0.38^{bc}	9.27 ± 0.37^{ab}	3.48 ± 0.07^{e}	3.35 ± 0.13^{d}	19.00 ± 0.76^{bc}	20.10 ± 0.32^{d}	99.45 ± 3.08^{d}	110.21 ± 3.09^{c}	0.72 ± 0.03^{n}	3.41 ± 0.08^{m}
A1	9.59 ± 0.39^{b}	9.44 ± 0.19^{a}	3.60 ± 0.06^{de}	3.46 ± 0.06^{d}	19.70 ± 0.43^{b}	24.50 ± 0.69^{a}	120.67 ± 2.05^{a}	126.73 ± 2.79^{ab}	5.89 ± 0.18^{h}	6.01 ± 0.24^{j}
Lim. fermentum										
10B	9.50 ± 0.28^{b}	9.60 ± 0.20^{a}	3.85 ± 0.19^{cd}	3.60 ± 0.10^{cd}	20.00 ± 0.70^{b}	22.00 ± 1.08^{bc}	89.21 ± 3.66^{ef}	95.87 ± 3.93^{d}	40.22 ± 1.33^{c}	47.32 ± 1.66^{c}
La5	9.46 ± 0.38^{bc}	9.62 ± 0.18^{a}	3.87 ± 0.17^{cd}	3.60 ± 0.014^{cd}	21.60 ± 1.73^{ab}	25.60 ± 1.18^{a}	89.87 ± 2.52^{e}	94.77 ± 2.08^{d}	39.98 ± 1.04^{c}	45.44 ± 1.41^{c}
F230	9.28 ± 0.24^{bc}	9.57 ± 0.22^{a}	3.66 ± 0.08^{d}	3.77 ± 0.17^{c}	22.10 ± 1.02^{a}	25.70 ± 0.67^{a}	94.56 ± 2.93^{e}	99.38 ± 4.57^{d}	35.43 ± 0.17^{d}	38.43 ± 0.81^{e}
DSM 20049	9.65 ± 0.29^{b}	9.59 ± 0.19^{a}	3.80 ± 0.18^{cd}	3.50 ± 0.07^{d}	18.40 ± 0.55^{c}	22.30 ± 0.89^{bc}	108.32 ± 2.17^{c}	112.20 ± 5.05^{c}	40.32 ± 0.81^{c}	42.21 ± 1.69^{d}
DSM 20052	9.28 ± 0.32^{bc}	9.08 ± 0.45^{ab}	4.00 ± 0.13^{cd}	3.66 ± 0.14^{cd}	14.90 ± 0.33^{e}	17.80 ± 0.43^{e}	61.57 ± 1.79^{g}	68.43 ± 1.92^{f}	24.54 ± 0.74^{e}	27.88 ± 0.61^{f}
P. pentosaceus										
I76	9.46 ± 0.21^{b}	9.12 ± 0.32^{ab}	3.90 ± 0.19^{cd}	3.77 ± 0.07^{c}	17.50 ± 0.63^{c}	19.80 ± 0.71^{de}	116.25 ± 4.42^{ab}	120.37 ± 4.94^{b}	3.87 ± 0.10^{k}	4.63 ± 0.09^{l}
T1C1	9.42 ± 0.26^{bc}	9.28 ± 0.20^{ab}	3.69 ± 0.18^{de}	3.59 ± 0.13^{cd}	16.70 ± 0.77^{d}	19.50 ± 0.94^{de}	112.29 ± 5.17^{b}	119.41 ± 3.22^{b}	11.34 ± 0.22^{f}	13.28 ± 0.48^{g}

CISEW5	9.42 ± 0.39^{bc}	9.23 ± 0.42^{ab}	3.74 ± 0.18^{cd}	3.60 ± 0.12^{cd}	18.60 ± 0.73^{b}	21.70 ± 0.61^{c}	117.35 ± 2.23^{ab}	126.91 ± 4.95^{ab}	4.32 ± 0.18^{j}	4.88 ± 0.11^{k}
13I	9.27 ± 0.38^{bc}	9.32 ± 0.21^{ab}	3.58 ± 0.08^{de}	3.50 ± 0.06^{d}	19.90 ± 0.38^{b}	25.70 ± 1.08^{a}	111.63 ± 3.35^{b}	118.87 ± 3.68^{b}	2.65 ± 0.11^{l}	3.05 ± 0.11^{n}
DSM 20336	9.41 ± 0.21^{b}	9,46 ± 0.37^{ab}	3.85 ± 0.10^{cd}	3.48 ± 0.15^{d}	23.30 ± 0.82^{a}	25.60 ± 0.72^{a}	121.27 ± 2.67^{a}	132.43 ± 2.91^{a}	4.69 ± 0.14^{i}	5.90 ± 0.11^{j}
Lev. brevis										
P7m1	9.01 ± 0.45^{bc}	9.00 ± 0.23^{b}	3.78 ± 0.19^{cd}	3.60 ± 0.15^{cd}	18.50 ± 0.39^{c}	22.30 ± 0.89^{bc}	104.60 ± 2.72^{cd}	112.32 ± 4.27^{bc}	59.66 ± 1.73^{a}	64.52 ± 2.65^{a}
MRS4-18	9.49 ± 0.43^{bc}	9.07 ± 0.45^{ab}	3.80 ± 0.14^{cd}	3.42 ± 0.08^{d}	18.40 ± 0.35^{c}	21.30 ± 0.85^{c}	88.85 ± 4.00^{ef}	94.65 ± 1.99^{d}	56.43 ± 2.26^{a}	60.05 ± 2.70^{a}
MCL-2	9.23 ± 0.18^{bc}	8.95 ± 0.44^{b}	3.80 ± 0.12^{cd}	3.61 ± 0.08^{c}	10.40 ± 0.21^{g}	15.80 ± 0.40^{f}	81.92 ± 3.77^{f}	86.32 ± 1.90^{e}	9.86 ± 0.28^{g}	10.11 ± 0.25^{h}
DSM 1269	9.33 ± 0.21^{bc}	9.33 ± 0.38^{ab}	4.06 ± 0.15^{c}	3.68 ± 0.11^{cd}	13.50 ± 0.59^{f}	18.50 ± 0.78^{e}	98.63 ± 3.25^{d}	109.53 ± 5.26^{c}	43.54 ± 1.35^{b}	49.96 ± 0.85^{b}
Controls										
SF	8.70 ± 0.43^{c}	8.90 ± 0.28^{b}	5.30 ± 0.19^{b}	5.00 ± 0.21^{b}	9.80 ± 0.41^{g}	10.30 ± 0.45^{g}	44.50 ± 0.89^{h}	46.51 ± 1.40^{g}	6.03 ± 0.25^{h}	6,98 ± 0.15^{i}
SFa	n.d.	n.d.	6.28 ± 0.17^{a}	6,21 ± 0.22^{a}	1.60 ± 0.07^{i}	2.00 ± 0.09^{i}	n.d.	n.d.	n.d.	n.d.

Fermentations were carried out with an initial cell density of 7 log CFU g^{-1} using strains of *Lactiplantibacillus plantarum* (1A7, CR1, M7S2, A1, DSM 1055), *Limosilactobacillus fermentum* (10B, La5, F230, DSM 20049, DSM 20052), *Pediococcus pentosaceus* (I76, T1C1, CISEW5, 13I, DSM 20336), and *Levilactobacillus brevis* (P7m1, MRS4, MCL-2, DSM 1269). UD indicates unfermented dough prepared using the same flour-to-water ratio (1:1), whereas SF refers to non-inoculated dough prepared with the same flour-to-water ratio and incubated under the same conditions as the inoculated doughs. SFa indicates additional doughs prepared using the same flour-to-water ratio (1:1), supplemented with chloramphenicol and cycloheximide (0.1 g L^{-1}), and incubated under the same conditions as the inoculated doughs. Values are expressed as mean ± standard deviation (n = 3), n.d., not detected. Different superscript letters (a–m) within the same column indicate statistically significant differences (ANOVA followed by Tukey's HSD test, p-value < 0.05); means sharing the same letter are not significantly different.

Table S3. Lactic acid bacteria (LAB) cell density and acidification parameters of doughs (flour:water ratio 1:1) prepared from wholegrain wheat flour obtained from naturally contaminated grain samples cultivated during the 2023–2024 growing season and fermented with selected LAB strains at 30 °C for 24 and 48 h.

	LAB (log CFU g^{-1})		pH		TTA (ml NaOH)		Lactic acid (mmol kg^{-1})		Acetic acid (mmol kg^{-1})	
UD	6.52 ± 0.21^{d}		6.33 ± 0.16^{a}		2.80 ± 0.09^{g-h}		n.d.		n.d.	
	24 h	48 h	24 h	48 h	24 h	48 h	24 h	48 h	24 h	48 h
Lp. plantarum										
DMS 1055	9.11 ± 0.23^{c}	8.86 ± 0.19^{b}	3.81 ± 0.08^{e}	3.67 ± 0.07^{cd}	25.20 ± 0.55^{a}	30.50 ± 0.58^{a}	124.86 ± 3.12^{cd}	131.94 ± 5.67^{bc}	4.01 ± 0.08^{k}	6.33 ± 1.84^{l}
1A7	9.49 ± 0.39^{bc}	9.89 ± 0.20^{a}	3.81 ± 0.07^{e}	3.41 ± 0.08^{d}	18.00 ± 0.54^{e}	22.30 ± 0.49^{d}	121.09 ± 2.54^{d}	128.74 ± 3.99^{c}	n.d.	n.d.
M7S2	9.94 ± 0.24^{a}	9.59 ± 0.28^{ab}	3.71 ± 0.18^{e}	3.28 ± 0.13^{d}	18.00 ± 0.49^{e}	24.10 ± 0.65^{c}	111.05 ± 4.66^{ef}	119.21 ± 3.10^{d}	n.d.	n.d.
CR1	9.10 ± 0.36^{c}	9.16 ± 0.28^{b}	3.87 ± 0.19^{de}	3.36 ± 0.11^{d}	18.00 ± 0.34^{e}	23.00 ± 0.87^{d}	116.79 ± 5.84^{de}	124.43 ± 4.11^{cd}	0.84 ± 0.03^{m}	4.14 ± 0.17^{m}
A1	9.61 ± 0.28^{c}	9.50 ± 0.39^{ab}	3.90 ± 0.09^{de}	3.46 ± 0.06^{d}	22.00 ± 1.03^{cd}	26.90 ± 1.10^{bc}	133.83 ± 6.16^{bc}	139.55 ± 2.51^{b}	7.39 ± 0.36^{i}	11.87 ± 0.38^{i}
Lim. fermentum										
10B	9.62 ± 0.19^{b}	9.48 ± 0.21^{a}	3.97 ± 0.07^{d}	3.68 ± 0.16^{cd}	21.20 ± 1.06^{cd}	26.10 ± 1.02^{bc}	91.49 ± 1.65^{h}	97.28 ± 1.95^{f}	50.45 ± 1.06^{c}	56.91 ± 1.20^{bc}
La5	9.55 ± 0.17^{b}	9.52 ± 0.18^{ab}	3.94 ± 0.12^{de}	3.75 ± 0.07^{c}	24.00 ± 0.60^{b}	28.00 ± 1.29^{b}	97.95 ± 4.21^{gh}	106.26 ± 2.02^{e}	52.14 ± 1.25^{c}	58.34 ± 1.17^{b}
F230	9.31 ± 0.18^{bc}	9.30 ± 0.42^{ab}	3.92 ± 0.10^{de}	3.59 ± 0.16^{cd}	25.60 ± 0.77^{a}	29.90 ± 1.32^{ab}	110.33 ± 4.41^{ef}	118.67 ± 4.03^{d}	40.10 ± 0.88^{e}	48.47 ± 1.36^{d}
DSM 20049	9.51 ± 0.43^{bc}	9.54 ± 0.29^{ab}	3.92 ± 0.14^{de}	3.73 ± 0.16^{cd}	21.60 ± 0.95^{cd}	26.70 ± 0.83^{bc}	121.63 ± 3.28^{cd}	129.44 ± 6.47^{c}	47.49 ± 1.61^{d}	52.31 ± 1.62^{c}
DSM 20052	8.97 ± 0.40^{c}	8.70 ± 0.37^{bc}	4.42 ± 0.18^{bc}	3.80 ± 0.08^{c}	17.60 ± 0.69^{e}	21.40 ± 1.07^{de}	78.04 ± 2.58^{i}	87.21 ± 1.66^{g}	30.61 ± 1.22^{f}	35.77 ± 1.40^{e}
P. pentosaceus										
I76	9.23 ± 0.29^{bc}	9.13 ± 0.36^{b}	3.97 ± 0.09^{de}	3.58 ± 0.15^{cd}	20.00 ± 0.98^{d}	22.00 ± 0.40^{d}	126.47 ± 2.40^{c}	135.75 ± 3.94^{bc}	5.07 ± 0.22^{j}	9.59 ± 0.39^{j}
T1C1	9.47 ± 0.29^{bc}	9.49 ± 0.32^{ab}	4.11 ± 0.16^{cd}	3.49 ± 0.12^{d}	20.00 ± 1.00^{d}	23.30 ± 0.91^{d}	125.22 ± 5.01^{c}	129.55 ± 5.57^{c}	14.99 ± 0.58^{g}	17.78 ± 0.55^{f}
CISEW5	9.51 ± 0.24^{bc}	9.40 ± 0.29^{ab}	4.05 ± 0.20^{cde}	3.52 ± 0.18^{cd}	21.20 ± 0.81^{d}	24.60 ± 0.71^{cd}	134.37 ± 3.22^{b}	142.44 ± 5.56^{ab}	5.07 ± 0.15^{j}	7.60 ± 0.24^{k}

13I	9.57 ± 0.32^{bc}	9.35 ± 0.47^{ab}	3.87 ± 0.19^{de}	3.54 ± 0.18^{cd}	22.80 ± 0.78^{bc}	28.50 ± 1.43^{ab}	125.94 ± 3.53^{cd}	131.20 ± 6.43^{bc}	3.17 ± 0.05^{l}	5.53 ± 0.19^{l}
DMS 20336	9.35 ± 0.44^{bc}	9.28 ± 0.45^{ab}	4.04 ± 0.20^{cde}	3.80 ± 0.11^{c}	21.70 ± 0.91^{c}	27.20 ± 0.60^{b}	147.46 ± 6.64^{a}	153.66 ± 6.45^{a}	7.60 ± 0.17^{i}	11.22 ± 0.49^{i}
Lev. brevis										
P7m1	9.05 ± 0.30^{c}	8.89 ± 0.36^{bc}	4.18 ± 0.15^{cd}	3.75 ± 0.18^{cd}	22.80 ± 0.64^{bc}	25.30 ± 1.04^{c}	116.25 ± 1.74^{e}	129.22 ± 5.30^{c}	66.70 ± 2.20^{a}	71.42 ± 2.14^{a}
MRS4-18	9.45 ± 0.32^{bc}	9.15 ± 0.27^{b}	4.30 ± 0.09^{c}	3.73 ± 0.07^{c}	20.80 ± 0.40^{d}	24.50 ± 0.49^{cd}	99.39 ± 3.88^{g}	108.90 ± 5.34^{de}	61.21 ± 1.71^{b}	69.78 ± 1.40^{a}
MCL-2	9.23 ± 0.23^{bc}	8.31 ± 0.31^{c}	4.80 ± 0.20^{b}	3.62 ± 0.16^{cd}	12.80 ± 0.62^{f}	16.10 ± 0.39^{f}	90.06 ± 3.96^{h}	98.11 ± 3.92^{f}	11.40 ± 0.22^{h}	16.23 ± 0.31^{g}
DSM 1269	8.94 ± 0.39^{c}	9.09 ± 0.45^{b}	4.42 ± 0.22^{bc}	3.73 ± 0.10^{c}	17.60 ± 0.37^{e}	20.60 ± 0.68^{e}	106.74 ± 3.20^{f}	123.09 ± 2.95^{cd}	50.66 ± 0.96^{c}	54.32 ± 2.28^{c}
Controls										
SF	8.87 ± 0.17^{c}	9.12 ± 0.34^{ab}	4.97 ± 0.21^{b}	4.40 ± 0.13^{b}	11.90 ± 0.38^{f}	13.70 ± 0.67^{g}	47.33 ± 1.66^{l}	53.22 ± 1.17^{h}	7.75 ± 0.77^{i}	14.18 ± 0.44^{h}
SFa	n.d.	n.d.	6.23 ± 0.16^{a}	6.20 ± 0.12^{a}	2.60 ± 0.11^{g}	2.90 ± 0.05^{h}	n.d.	n.d.	n.d.	n.d.

Fermentations were carried out with an initial cell density of 7 log CFU g^{-1} using strains of *Lactiplantibacillus plantarum* (1A7, CR1, M7S2, A1, DSM 1055), *Limosilactobacillus fermentum* (10B, La5, F230, DSM 20049, DSM 20052), *Pediococcus pentosaceus* (I76, T1C1, CISEW5, 13I, DSM 20336), and *Levilactobacillus brevis* (P7m1, MRS4, MCL-2, DSM 1269). UD indicates unfermented dough prepared using the same flour-to-water ratio (1:1), whereas SF refers to non-inoculated dough prepared with the same flour-to-water ratio and incubated under the same conditions as the inoculated doughs. SFa indicates additional doughs prepared using the same flour-to-water ratio (1:1), supplemented with chloramphenicol and cycloheximide (0.1 g L^{-1}), and incubated under the same conditions as the inoculated doughs. Values are expressed as mean ± standard deviation (n = 3). Different superscript letters (a–m) within the same column indicate statistically significant differences (ANOVA followed by Tukey's HSD test, p-value < 0.05); means sharing the same letter are not significantly different.

Table S4. Lactic acid bacteria (LAB) cell density and acidification parameters of doughs (flour:water ratio 1:1) prepared from wholegrain wheat flour obtained from naturally contaminated grain samples cultivated during the 2023–2024 growing season and fermented with selected LAB strains at 37 °C for 24 and 48 h.

	LAB (log CFU g^{-1})		pH		TTA (ml NaOH)		Lactic acid (mmol kg^{-1})		Acetic acid (mmol kg^{-1})	
UD	6.52 ± 0.21^{d}		6.33 ± 0.16^{a}		2.80 ± 0.09^{g}		n.d.		n.d.	
	24 h	48 h	24 h	48 h	24 h	48 h	24 h	48 h	24 h	48 h
Lp. plantarum										
DMS 1055	8.48 ± 0.20^{b}	8.23 ± 0.18^{c}	3.88 ± 0.09^{d}	3.83 ± 0.13^{cd}	26.00 ± 0.81^{a}	32.10 ± 0.93^{a}	125.66 ± 2.64^{c}	133.01 ± 4.12^{cd}	4.60 ± 0.14^{j}	6.79 ± 0.20^{k}
1A7	9.76 ± 0.47^{a}	8.30 ± 0.29^{c}	3.58 ± 0.17^{e}	3.51 ± 0.09^{d}	18.40 ± 0.52^{d}	23.00 ± 0.81^{ef}	122.17 ± 3.67^{cd}	129.91 ± 4.55^{cd}	n.d.	n.d.
M7S2	9.54 ± 0.18^{a}	8.61 ± 0.27^{bc}	3.59 ± 0.10^{e}	3.44 ± 0.06^{e}	18.50 ± 0.57^{d}	24.90 ± 0.57^{de}	112.16 ± 2.24^{de}	120.13 ± 2.64^{d}	n.d.	n.d.
CR1	9.32 ± 0.17^{ab}	9.10 ± 0.32^{ab}	3.55 ± 0.12^{e}	3.49 ± 0.07^{d}	18.50 ± 0.76^{d}	23.90 ± 0.53^{e}	117.80 ± 4.48^{d}	125.14 ± 5.63^{d}	1.01 ± 0.05^{l}	4.63 ± 0.10^{m}
A1	9.70 ± 0.26^{a}	9.33 ± 0.37^{a}	3.72 ± 0.12^{de}	3.63 ± 0.18^{cd}	22.40 ± 0.67^{b}	25.20 ± 0.87^{c}	134.21 ± 3.62^{b}	140.33 ± 3.51^{bc}	7.92 ± 0.23^{h}	12.12 ± 0.44^{h}
Lim. fermentun										
10B	9.50 ± 0.27^{a}	8.80 ± 0.30^{b}	3.98 ± 0.16^{cd}	3.73 ± 0.09^{cd}	21.90 ± 0.70^{bc}	27.00 ± 1.11^{cd}	92.62 ± 3.80^{f}	98.13 ± 2.85^{f}	50.90 ± 1.12^{c}	57.30 ± 1.32^{b}
La5	9.29 ± 0.18^{ab}	9.26 ± 0.19^{a}	3.97 ± 0.19^{cd}	3.91 ± 0.11^{c}	24.60 ± 1.01^{ab}	28.80 ± 0.92^{bc}	98.92 ± 2.67^{e}	107.04 ± 4.39^{e}	53.04 ± 1.91^{c}	59.99 ± 2.70^{b}
F230	9.39 ± 0.38^{ab}	8.83 ± 0.36^{ab}	3.91 ± 0.19^{de}	3.78 ± 0.09^{cd}	26.00 ± 0.57^{a}	30.50 ± 1.07^{ab}	111.12 ± 2.89^{de}	119.91 ± 4.20^{d}	40.99 ± 1.43^{d}	49.52 ± 0.84^{d}
DSM 20049	9.40 ± 0.25^{ab}	9.29 ± 0.198^{a}	3.75 ± 0.07^{de}	3.70 ± 0.11^{c}	21.80 ± 0.65^{bc}	27.00 ± 0.54^{c}	122.72 ± 2.45^{cd}	130.12 ± 3.90^{cd}	49.92 ± 1.50^{c}	54.11 ± 1.08^{c}
DSM 20052	9.07 ± 0.41^{ab}	8.28 ± 0.24^{c}	4.17 ± 0.19^{cd}	3.98 ± 0.18^{bc}	18.40 ± 0.83^{d}	22.60 ± 0.79^{ef}	79.10 ± 2.61^{g}	88.91 ± 1.96^{g}	32.12 ± 0.71^{e}	37.81 ± 1.29^{e}
P. pentosaceus										
I76	9.19 ± 0.34^{ab}	8.30 ± 0.14^{c}	3.83 ± 0.15^{de}	3.71 ± 0.15^{cd}	20.80 ± 0.67^{c}	22.40 ± 0.63^{f}	127.12 ± 5.21^{bc}	136.16 ± 3.00^{c}	5.70 ± 0.11^{i}	11.12 ± 0.39^{i}
T1C1	9.41 ± 0.36^{ab}	8.69 ± 0.19^{b}	3.83 ± 0.08^{d}	3.78 ± 0.14^{cd}	20.80 ± 0.85^{c}	23.90 ± 0.45^{e}	126.12 ± 5.04^{cd}	130.14 ± 2.47^{cd}	15.82 ± 0.47^{f}	18.14 ± 0.74^{f}
CISEW5	9.21 ± 0.31^{ab}	8.82 ± 0.26^{ab}	3.69 ± 0.17^{de}	3.62 ± 0.18^{cd}	21.90 ± 0.64^{bc}	25.10 ± 1.03^{de}	135.41 ± 3.79^{b}	143.18 ± 6.59^{ab}	5.67 ± 0.26^{i}	8.12 ± 0.18^{j}

13I	9.48 ± 0.29^{a}	8.13 ± 0.35^{c}	3.42 ± 0.14^{e}	3.38 ± 0.10^{e}	23.30 ± 0.96^{b}	29.60 ± 0.74^{b}	126.44 ± 2.66^{c}	132.11 ± 6.08^{cd}	3.66 ± 0.15^{k}	5.98 ± 0.19^{l}
DMS 20336	9.29 ± 0.33^{sb}	8.21 ± 0.20^{c}	3.75 ± 0.09^{de}	3.61 ± 0.10^{d}	22.50 ± 0.56^{b}	28.00 ± 1.15^{bc}	148.04 ± 3.26^{a}	154.12 ± 5.55^{a}	7.91 ± 0.27^{h}	13.01 ± 0.53^{h}
Lev. brevis										
P7m1	8.77 ± 0.39^{b}	8.74 ± 0.34^{bc}	3.89 ± 0.07^{d}	3.76 ± 0.09^{cd}	23.00 ± 0.51^{b}	25.90 ± 0.73^{cd}	116.16 ± 3.60^{d}	139.91 ± 2.94^{b}	67.20 ± 2.22^{a}	74.72 ± 2.69^{a}
MRS4-18	9.34 ± 0.42^{ab}	8.29 ± 0.22^{a}	3.63 ± 0.09^{e}	3.57 ± 0.06^{d}	21.10 ± 0.84^{bc}	25.00 ± 0.50^{d}	100.01 ± 4.10^{ef}	109.12 ± 4.26^{e}	62.04 ± 1.43^{b}	71.10 ± 2.92^{a}
MCL-2	9.14 ± 0.30^{ab}	9.54 ± 0.31^{a}	3.82 ± 0.15^{de}	3.61 ± 0.08^{d}	13.00 ± 0.36^{e}	17.10 ± 0.50^{g}	91.17 ± 3.28^{f}	99.43 ± 1.89^{f}	12.11 ± 0.36^{g}	18.07 ± 0.49^{f}
DSM 1269	9.14 ± 0.41^{ab}	8.70 ± 0.15^{b}	4.26 ± 0.12^{c}	4.10 ± 0.11^{bc}	18.10 ± 0.74^{d}	21.20 ± 0.93^{f}	107.13 ± 5.25^{e}	124.04 ± 4.96^{d}	51.12 ± 2.10^{c}	56.04 ± 1.40^{bc}
Controls										
SF	8.41 ± 0.32^{b}	8.22 ± 0.23^{c}	4.95 ± 0.09^{b}	4.37 ± 0.16^{b}	12.00 ± 0.46^{f}	14.10 ± 0.58^{h}	48.12 ± 1.06^{h}	54.71 ± 1.59^{h}	8.05 ± 0.35^{h}	15.15 ± 0.32^{g}
SFa	n.d.	n.d.	6.21 ± 0.15^{a}	6.19 ± 0.13^{a}	2.70 ± 0.05^{g}	3.10 ± 0.12^{i}	n.d.	n.d.	n.d.	n.d.

Fermentations were carried out with an initial cell density of 7 log CFU g^{-1} using strains of *Lactiplantibacillus plantarum* (1A7, CR1, M7S2, A1, DSM 1055), *Limosilactobacillus fermentum* (10B, La5, F230, DSM 20049, DSM 20052), *Pediococcus pentosaceus* (I76, T1C1, CISEW5, 13I, DSM 20336), and *Levilactobacillus brevis* (P7m1, MRS4, MCL-2, DSM 1269). UD indicates unfermented dough prepared using the same flour-to-water ratio (1:1), whereas SF refers to non-inoculated dough prepared with the same flour-to-water ratio and incubated under the same conditions as the inoculated doughs. SFa indicates additional doughs prepared using the same flour-to-water ratio (1:1), supplemented with chloramphenicol and cycloheximide (0.1 g L^{-1}), and incubated under the same conditions as the inoculated doughs. Values are expressed as mean ± standard deviation (n = 3). Different superscript letters (a–n) within the same column indicate statistically significant differences (ANOVA followed by Tukey's HSD test, p-value < 0.05); means sharing the same letter are not significantly different.

.

.

Table S5 Key physicochemical properties of the detected mycotoxins identified in wholegrain wheat flour. Data extracted from ChemSpider (Royal Society of Chemistry)

Mycotoxin	Chemical structure	MW (g mol^{-1})	MV (cm^3 mol^{-1})	LogD (pH 5.5)
Deoxynivalenol (DON)	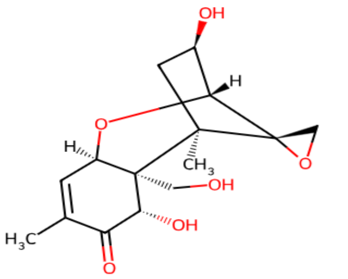	296.32	199.4	-0.88
Deoxynivalenol-3-glucoside (DON-3-G)	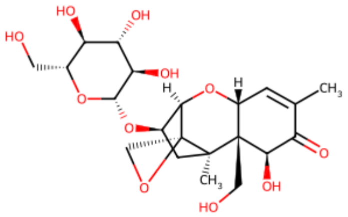	458.46	284.7	-2.92
3-Acetyl-deoxynivalenol (3-ADON)	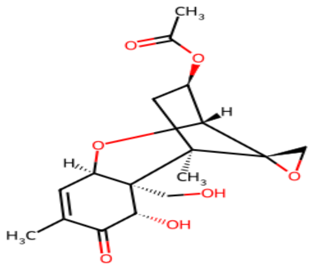	338.36	237.5	-0.28

Enniatin B_1 (ENN B1)	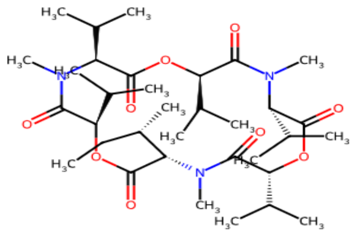	653.86	633.8	3.81
Moniliformin (MON)	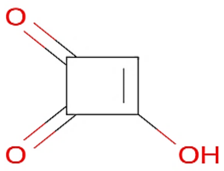	98.06	52.2	-3.79
Enniatin B (ENN B)	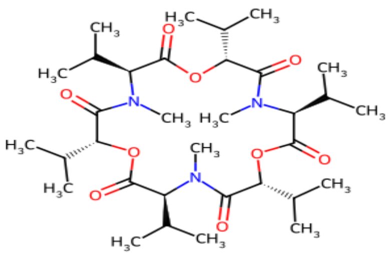	639.83	617.2	3.42

MW, Molecular weight; MV, molecular volume; LogD (pH 5.5), distribution coefficient (octanol/water) at specific pH.
